# Hemorrhoidal Disease in the Diabetic Population: The Effects of Glucose Regulation and Lipid Profile

**DOI:** 10.3390/life15020178

**Published:** 2025-01-25

**Authors:** Enver Ciftel, Sedat Ciftel, Serpil Ciftel, Filiz Mercantepe, Remzi Adnan Akdogan

**Affiliations:** 1 Department of Endocrinology and Metabolism, Sivas Numune Hospital, Sivas 58040, Türkiye; enver.ciftel@saglik.gov.tr; 2 Department of Gastroenterology and Hepatology, Erzurum Training and Research Hospital, Erzurum 5240, Türkiye; sedat.ciftel@saglik.gov.tr; 3 Department of Endocrinology and Metabolism, Erzurum Training and Research Hospital, Erzurum 5240, Türkiye; serpil.ciftel@saglik.gov.tr; 4 Department of Endocrinology and Metabolism, Recep Tayyip Erdogan University, Rize 53100, Türkiye; 5 Department of Gastroenterology and Hepatology, Recep Tayyip Erdogan University, Rize 53100, Türkiye

**Keywords:** hemorrhoidal disease, diabetes mellitus, glycemic control, lipid profile, dyslipidemia, hypertension

## Abstract

Background: Hemorrhoidal disease is a common anorectal condition characterized by the enlargement and distal displacement of the typical vascular structures in the anal canal. The relationship between DM, lipid metabolism, and hemorrhoidal disease remains underexplored. This study aims to investigate the prevalence of hemorrhoids and the association between glycemic control and lipid profile in diabetic patients. Methods: This retrospective cross-sectional study included 752 patients who underwent colonoscopy at Erzurum Regional Training and Research Hospital between June 2021 and August 2024. The study population comprised 452 patients with type 2 diabetes mellitus (mean age 63.4 ± 11.0) and 300 nondiabetic patients (mean age 62.8 ± 10.8). The presence of hemorrhoids was confirmed through colonoscopy. Glycemic control parameters, lipid profile, and other biochemical parameters were analyzed. Results: Hemorrhoids were found in 47.3% (*n* = 214) of diabetic patients and 17.3% (*n* = 52) of nondiabetic patients, indicating a significantly higher prevalence in the diabetic group (OR = 4.3, CI = 3.0–6.2, *p* < 0.001). Diabetic patients with hemorrhoids had significantly higher mean HbA1C (8.1 ± 2.1 vs. 7.5 ± 1.8, *p* < 0.001), low-density lipoprotein (*p* < 0.001), and triglyceride levels (*p* = 0.005) compared to those without hemorrhoids. Additionally, a longer duration of diabetes and higher hypertension prevalence were observed in the hemorrhoid group. Conclusions: The findings suggest that poor glycemic control and dyslipidemia are significantly associated with an increased prevalence of hemorrhoids in diabetic patients. These results highlight the importance of comprehensive management of diabetes, including lipid control, to potentially reduce the risk of hemorrhoidal disease.

## 1. Introduction

Hemorrhoidal disease represents a prevalent anorectal condition that arises from the degeneration of the vascular frameworks and connective tissues within the anal canal, which are integral to the processes of defecation and anal continence. The precise prevalence remains indeterminate, as individuals typically refrain from seeking medical intervention until the condition becomes intolerable, often influenced by personal and cultural inhibitions stemming from feelings of embarrassment. Epidemiological investigations reveal a prevalence rate ranging from approximately 4.4% to 38%, with a notable peak occurring within the demographic of individuals aged 45 to 65 years [1,2].

The dentate line is important in the classification of hemorrhoids. Internal hemorrhoids, which are covered with mucosa, develop proximal to the dentate line. External hemorrhoids, which are covered with squamous epithelium, originate distal to the dentate line. In some patients, internal and external hemorrhoids may coexist [3]. The pathogenesis of hemorrhoids is complex and is influenced by several factors, including age, diet, obesity, constipation, diarrhea, and other conditions that increase intra-abdominal pressure. The exact cause is unknown, but conditions that increase intra-abdominal pressure can increase the pressure in the hemorrhoidal venous plexus and accelerate its development. These include straining during constipation, chronic diarrhea, irritable bowel syndrome, pregnancy, childbirth, obesity, lack of exercise, low-fiber diets, smoking, anal intercourse, prolonged standing, cirrhosis with ascites, pelvic floor dysfunction, and chronic cough [4]. In recent years, there has been increasing interest in the possible links between metabolic and vascular disorders and the development of hemorrhoids.

Diabetes mellitus (DM) is a disease characterized by chronic hyperglycemia and various metabolic disorders, and its prevalence is rapidly increasing worldwide. Its prevalence is expected to grow to 783 million by 2045 [5]. DM is associated with many systemic complications, which are closely linked to vascular and inflammatory processes. Microvascular and macrovascular complications seen in diabetic patients are related to various factors such as vascular inflammation, endothelial dysfunction, and oxidative stress [6]. These systemic effects of DM and the antidiabetic treatments can affect many organs and tissues, including the gastrointestinal system. In addition to metabolic and vascular factors, gastrointestinal disorders are prevalent in diabetic patients and may contribute to the pathogenesis of hemorrhoidal disease. Chronic constipation, reported in 25–30% of diabetic patients, and diabetic diarrhea, affecting 10–20% of this population, are among the most common contributors [7,8,9,10]. Irregular bowel habits have been reported in 25.8% of diabetic patients [9]. These conditions, combined with motility disorders such as gastroparesis [11], can exacerbate anorectal pressure and predispose individuals to hemorrhoidal disease. The risk of anorectal diseases such as hemorrhoids may increase with chronic complications of diabetes on intestinal motility or weight gain. Coexisting gastrointestinal disorders contribute to increased morbidity by negatively affecting the already impaired quality of life in diabetic patients, both through poor glycemic control and its complications. Diabetic autonomic neuropathy, especially that resulting from long-standing diabetes, can lead to gastrointestinal system (GIS) hyperfunction or hypofunction, delayed gastric and esophageal emptying, diabetic gastroparesis, gastroesophageal reflux, diarrhea, and constipation [12].

Our aim in this study is to investigate the prevalence of hemorrhoids in diabetic patients and whether glycemic control, lipid profile, and antidiabetic treatments are related to hemorrhoids, thus contributing to the development of new approaches in the treatment of both diabetes and hemorrhoids.

## 2. Patients and Methods

This retrospective cross-sectional study included patients undergoing colonoscopy from June 2021 to August 2024. A total of 752 participants were included: 452 patients with type 2 diabetes (230 male, 222 female, mean age 63.4 ± 11.0) and 300 nondiabetic patients (148 female, 152 male, mean age 62.8 ± 10.8) who applied to Erzurum Regional Education and Research Hospital with current lower GI complaints and underwent colonoscopy. The study was conducted according to the Declaration of Helsinki and approved by the Local Ethics Committee of Erzurum Training and Research Hospital (ethical approval no: E-40465587-050.01.04-1041, 2024/92). Since it had a retrospective design, informed consent was not obtained from the participants for the study. However, an informed consent form was obtained from all participants for the colonoscopy procedure, which is mandatory before every invasive procedure.

Hemorrhoids were detected in 214 of the 452 patients with diabetes, and 238 patients had normal colonoscopic findings. The type and grading of hemorrhoids were performed according to the current literature [4]. The patients were asked to fill out a questionnaire before the colonoscopy that questioned their current symptoms, duration of diabetes, and antidiabetic treatments. The patients’ hemogram and biochemical blood analyses were performed simultaneously with the colonoscopy. Fasting blood glucose (FBG), glycosylated hemoglobin (HbA1C), low-density lipoprotein (LDL), high-density lipoprotein (HDL), triglyceride (TG), hemoglobin (Hgb), and ferritin levels were analyzed.

Exclusion criteria: patients with type 1 diabetes, pregnant women, those with disorders related to the gastrointestinal system, and patients with a disease other than hemorrhoids detected during colonoscopy were not included in the study.

### Statistical Analysis

Analyses were performed using the IBM SPSS 22 statistical analysis program. After the Kolmogorov–Smirnov test and skewness and kurtosis were evaluated to determine whether the data were normally distributed, Student’s *T* test or the Mann–Whitney U test was used for the differences in parameters between two groups, and One-Way ANOVA or Kruskal–Wallis tests were used for comparisons of more than two groups. Post hoc tests after the ANOVA test were performed using the Bonferroni test when variances were homogeneous and Tamhane’s T2 test was used when variances were not homogeneous. In 2 × 2 comparisons between categorical variables, the Pearson Chi-square test was used if the expected value was >5, the Yates Chi-square test was used if the expected value was 3–5, and Fisher’s exact test was used if the expected value was <3. In comparisons greater than 2 × 2 between categorical variables, the Pearson Chi-square test was used when the expected value was >5, and the Fisher–Freeman–Halton test was used when the expected value was <5. When comparing two quantitative variables, the Spearman correlation test was used.

## 3. Results

The pre-colonoscopy symptoms of all patients (*n* = 752) in this study are evaluated in Table 1. In total, 46.8% of the patients had chronic constipation, 29.2% had rectal bleeding, 11.8% had chronic diarrhea, and 9.57% had weight loss symptoms.

Table 2 compares patients in the diabetic and nondiabetic groups in terms of hemorrhoid frequency. While the hemorrhoid frequency in the diabetic group (*n* = 452) was 47.3% (*n* = 214), it was 17.3% (*n* = 52) in the nondiabetic group (*n* = 300). The hemorrhoid frequency in the diabetic group was 4.3 times higher than in the nondiabetic group (OR = 4.3, CI = 3.0–6.2, *p* < 0.001). Glomerular filtration rate (eGFR) and Hgb were significantly lower in the diabetic group than in the nondiabetic group (*p* < 0.001).

In Table 3, all diabetic patients are divided into two groups: those with hemorrhoids (*n* = 214) and those without (*n* = 238). These groups were compared in terms of age, gender, duration of diabetes, and antidiabetic treatments used. No difference was found between the two groups in terms of age, gender, antidiabetic treatments used, and metformin use. The duration of diabetes in the hemorrhoid group was significantly longer than in the non-hemorrhoid group. The mean duration of diabetes in the hemorrhoid group was 13.1 ± 6.9 years, while it was 7.48 ± 4.4 years in the non-hemorrhoid group (*p* < 0.001). Hypertension (HT) frequency in the hemorrhoid group was significantly higher than in the non-hemorrhoid group. The frequency of HT in the hemorrhoid group was 72.9%, while it was 58.4% in the non-hemorrhoid group (*p* = 0.002).

In Table 4, the diabetic groups were compared regarding biochemical and hematological parameters. The hemorrhoid group had significantly higher HbA1C (8.1 ± 2.1) (*p* = 0.001). In addition, the hemorrhoid group had significantly higher LDL and TG levels (respectively, *p* = 0.001, *p* = 0.005). There was no difference between the groups regarding eGFR, serum creatinine, Hgb, ferritin, uric acid, and HDL.

Table 5 shows the distribution of hemorrhoid types in patients in the hemorrhoid group and compares them according to hemorrhoid types in terms of age, diabetes duration, and blood glucose regulation. Internal hemorrhoids were seen in 77.6% of the patients (*n* = 166), external hemorrhoids in 15.9% (*n* = 34), and internal and external hemorrhoids in 6.5% (*n* = 14). The mean age of the external hemorrhoid group (68.7 ± 10.6) was significantly higher than that of the internal hemorrhoids (62.1 ± 10.8) (*p* = 0.005). There was a difference between all groups in terms of diabetes duration (*p* < 0.001). The mean diabetes duration of the external + internal hemorrhoid group was 23 ± 4.8 years, that of the internal hemorrhoid group was 12.9 ± 6.6 years, and that of the external hemorrhoid group was 10.0 ± 4.8 years. The groups with differences in blood glucose regulation were determined as internal and external hemorrhoid groups. The mean HbA1C (8.8 ± 1.69) and FBG (172(86–357)) in the external hemorrhoid group were found to be statistically higher than the internal hemorrhoid group (*p* < 0.001, *p* < 0.001, respectively).

The One-Way ANOVA test was used to determine whether there were differences in age, diabetes duration, and HbA1c levels among hemorrhoid types in diabetic patients. There was a difference between the types of hemorrhoids in terms of patient age (*p* = 0.005). Since the variables for age were homogeneous, the groups with differences were determined as internal hemorrhoids and external hemorrhoids using the post hoc Bonferroni test (*p* = 0.004).

Differences were found between hemorrhoid types in terms of diabetes duration (*p* < 0.001) and the difference was determined among all groups using the post hoc Tamhane test (*p* = 0.015 for internal–external, *p* < 0.001 for internal–internal and external, and *p* < 0.001 for external–internal and external).

Differences between types of hemorrhoids were detected in terms of HbA1c (*p* < 0.001) and groups with differences were identified using the post hoc Bonferroni test (*p* = 0.025 for internal–external, *p* = 0.001 for internal–internal and external).

Spearman correlation analysis was performed to evaluate the relationship between internal hemorrhoid grades and clinical/biochemical parameters, including HbA1C, glucose, and lipid levels. Diabetic patients with internal hemorrhoids were compared in terms of internal hemorrhoid grade and the parameters given in Table 6. A strong positive correlation was found between diabetes duration and internal hemorrhoid grade (r = 0.515, R^2^ = 0.311, *p* < 0.001). In addition, a negative, weak, but significant correlation was found between ferritin level and internal hemorrhoid grade (r = −0.172, *p* = 0.021). No difference was found between HbA1C, glucose, lipid parameters, Hgb, and internal hemorrhoid grades.

## 4. Discussion

Hemorrhoidal disease is a mucosal cushion consisting of connective tissue, muscle fibers, and veins that play a role in defecation and anal continence. Due to many reasons, such as constipation, diarrhea, advancing age, diet, obesity, and increased intra-abdominal pressure, the venous pressure that increases with straining relaxes the fibers and expands the cushions is very important in pathogenesis. In diabetic patients, bowel movements are impaired, and lower GI symptoms such as chronic diarrhea and chronic constipation are seen much more frequently than in nondiabetic individuals [9,13]. This study aimed to investigate the prevalence of hemorrhoids in diabetic and nondiabetic patients and the relationship between the presence of hemorrhoids in diabetic patients and various clinical and biochemical parameters such as diabetes duration, hypertension frequency, HbA1C levels, and blood glucose regulation. In our study, hemorrhoids were detected in 47.3% of diabetic patients, which is relatively high compared to the prevalence of hemorrhoids in the general population. In a prevalence study conducted by Parvez Sheikh et al. including 16,015 participants, the prevalence of hemorrhoids was found to be 11% in the general population [14]. The occurrence of hemorrhoids exhibits a heightened frequency among specific demographics, particularly among pregnant or postpartum women, with prevalence rates ranging from 12% to 41% within these populations [15,16]. Nevertheless, the prevalence of hemorrhoids identified in diabetic patients in our investigation surpasses these figures significantly. This finding suggests the potential effect of diabetes on the development of hemorrhoids. The frequency of hemorrhoids in nondiabetic patients was found to be 17.3%. There may be several mechanisms underlying the fact that the frequency of hemorrhoids was found to be 4.3 times higher in diabetic patients compared to nondiabetic patients in our study. Chronic diarrhea and constipation are the most common lower GI symptoms in diabetic patients, and the most common lower GI symptoms in diabetic patients participating in our study were constipation in 50% and chronic diarrhea, detected in 10.8%. This situation is consistent with studies investigating GI complaints in diabetic patients [17,18]. A significant relationship was found between the duration of diabetes and the presence and the grade of hemorrhoids. The duration of diabetes in patients with hemorrhoids was significantly longer than in the non-hemorrhoid group. This finding suggests that long-term diabetes may contribute to the development of hemorrhoids through many possible mechanisms. In symptom-based studies, it has been reported that lower GI symptoms, especially constipation, are associated with the duration of diabetes [19]. The chronic inflammatory nature of diabetes, the increase in intra-abdominal pressure with GIS symptoms, and the microvascular and macrovascular complications may be some of the underlying causes. In addition, the fact that we found a higher frequency of HT in the hemorrhoid group supports the role of vascular factors in the development of hemorrhoids. HT causes structural changes in the arterial and venous systems, and these changes may affect the vascular structures in the anus and rectal region and create a basis for the development of hemorrhoids. In our study, diabetic patients with hemorrhoids had worse lipid profiles with high triglyceride and LDL, and lipids may contribute to vascular inflammation and endothelial dysfunction, causing hemorrhoidal veins to dilate and become chronic. In addition, a high-fat diet and subsequent obesity may predispose to hemorrhoids due to increased stress on the rectal muscle. Indeed, the fact that coronary heart disease is more common in people with hemorrhoids in some studies supports our findings [20]. Hemorrhoids and varicose veins are aneurysms of the venous vascular system with common pathophysiology, and it is accepted that their pathophysiology may show similarities with atherosclerotic vascular disease, in which dyslipidemia is a significant risk factor [21].

GIS symptoms that diabetic patients frequently suffer may be a sign of poor glycemic control and diabetic complications [22]. In our study, HbA1C levels were significantly higher in the hemorrhoid group than in the non-hemorrhoid group. Since HbA1C levels are a long-term indicator of blood sugar control, these findings suggest that poor glycemic control may be an essential factor in the development of hemorrhoids.

Other underlying mechanisms for diabetic gastrointestinal symptoms may include autonomic and peripheral neuropathy, structural and functional central nervous system changes (diabetic encephalopathy), acute and chronic dysglycemia, psychological dysfunction, and antidiabetic treatments [13]. The most common gastrointestinal complaints related to antidiabetic treatments are nausea, vomiting, postprandial fullness due to GLP-1 receptor agonists, abdominal pain, discomfort, diarrhea due to metformin, diarrhea, gas, and bloating due to alpha-glucosidase inhibitors and in our study, no difference was observed between the groups in terms of oral antidiabetic drugs and insulin use [13]. Some studies have reported that metformin use in type 2 diabetic patients may have a protective role against the development of hemorrhoids and reduce the risk of varicose veins [23,24]. In our study, in line with these studies, metformin use was less frequent in the hemorrhoid group, but no statistical difference was obtained.

In our study, significant differences were also observed between hemorrhoid types regarding diabetes duration and glucose regulation. The most prolonged duration of diabetes was found in the external and internal hemorrhoid group, while the highest HbA1C and fasting glucose levels were found in the external hemorrhoid group. Especially in the external hemorrhoid group, which is known to be more painful, the higher HbA1C levels and fasting glucose levels indicate that glycemic control is inadequate in these patients. This finding suggests that poor glycemic control may increase the severity of hemorrhoids, and severe anorectal symptoms may impair the quality of life of patients and cause poor glycemic control. Since more severe hemorrhoid types may be associated with poorly regulated diabetes, it is indispensable to question all diabetic patients, especially those with a long duration of diabetes, in terms of anorectal symptoms.

The negative correlation found between ferritin level and internal hemorrhoid grade suggests that iron metabolism may be affected in chronic diseases, which may play a role in the development of hemorrhoids. However, since hemoglobin did not support this finding, we believe that further research with larger numbers of participants is needed on its clinical significance and potential mechanisms.

More research should be conducted on how lower gastrointestinal complaints in diabetic patients affect their daily lives and psychological status. Our findings may contribute to the development of more holistic approaches in the management of hemorrhoids and diabetes. The effects of lifestyle changes such as diet and exercise on both glycemic control and hemorrhoid development should not be ignored. It should also be noted that exercise can reduce the risk of hemorrhoids by increasing blood circulation and preventing constipation. Suggestions such as increasing the fiber intake recommended in the diet for hemorrhoids are also helpful suggestions in a diabetic diet, where GI involvement is frequently seen [25]. Raising the awareness of diabetic patients about lifestyle adjustments is also very important in terms of its protective effect against the development of hemorrhoidal disease. In addition, detection of hemorrhoidal disease and treatments for hemorrhoidal disease may be indirectly beneficial in blood sugar regulation.

Our study is the first and only study in the literature investigating the relationship between diabetes and hemorrhoids. The novel findings of our study highlight the close association between diabetes, poor glycemic control, dyslipidemia, and the prevalence of hemorrhoidal disease. These results suggest that addressing glycemic and lipid control in diabetic patients could have a dual benefit: improving glycemic management and potentially reducing the burden of hemorrhoidal disease. Future research should focus on prospective studies to validate these findings and explore targeted interventions for this comorbidity. Other strengths of our study include the following: Since hemorrhoids are a disease closely associated with advanced age and male gender, the fact that the mean age and gender distribution of the groups compared in our study were equal is very important in terms of the reliability of the results by preventing the negative effects of confounding factors. In addition, it is known that antidiabetic treatments used, as well as diabetes itself, have negative effects on GIS symptoms. In our study, no difference was observed in terms of the antidiabetic treatments received by the patients. The limitations of our study are that it was conducted in a single center retrospectively, and the sample size was insufficient, so specific analyses could not be performed for internal hemorrhoid grades. Although our investigation revealed noteworthy correlations between the duration of diabetes, poor glycemic regulation, and the incidence of hemorrhoidal disease, it is crucial to acknowledge that hemorrhoidal disease arises from multiple etiological factors. Variables such as dietary patterns, levels of physical activity, body mass index, gastrointestinal disorders (including chronic constipation or diarrhea), and additional comorbidities such as hypertension may also contribute to its pathophysiology. Unfortunately, all these variables were not evaluated due to the retrospective design nature of the present study. In addition, the oral antidiabetic groups used were not specified separately except for metformin. Furthermore, our investigation evaluated the management of diabetes through the analysis of HbA1c levels within the framework of our study. Nevertheless, it is crucial to acknowledge that effective diabetes management necessitates a comprehensive approach, which encompasses lifestyle modifications, adherence to prescribed pharmacotherapy, consistent monitoring of glycemic levels, and the management of concomitant health conditions. The design of our study was cross-sectional, thereby imposing certain limitations in establishing causal relationships and delivering an exhaustive evaluation of diabetes management practices. Nonetheless, as the inaugural study in the existing literature to explore this particular association, it illuminates a novel domain of inquiry and lays the groundwork for subsequent research endeavors. We posit that our results will catalyze more in-depth examinations into the interplay between diabetes management and hemorrhoidal disease, thereby aiding in the formulation of more efficacious management strategies. A notable limitation of our research is the potential for selection bias that may arise from the inclusion of patients undergoing colonoscopy, rather than a population-based sampling of individuals diagnosed with diabetes mellitus. This methodological choice may lead to an over-representation of gastrointestinal disorders, including hemorrhoidal conditions, within our study cohort. Consequently, the prevalence rates recorded in this investigation should be approached with caution, as they may lack generalizability to the broader diabetic population. Nonetheless, the substantial sample size and the noteworthy association (OR 4.3) identified between diabetes and hemorrhoidal disease lend credence to the validity of our conclusions. Future investigations should prioritize population-based methodologies to mitigate selection bias and further substantiate these findings.

## 5. Conclusions

The presence of hemorrhoids in diabetic patients is closely associated with long-term diabetes and poor glycemic control. In the management of diabetes, glycemic control is important not only in terms of preventing the complications of diabetes but also in terms of preventing or managing the development of hemorrhoids. The fact that lower GI complaints, which are considered embarrassing for patients, are not expressed unless specifically asked by the doctor may be one of the underlying reasons for the failure to achieve glycemic regulation. Since our study is the first to examine the relationship between diabetes and hemorrhoids, we believe it fills an important gap in the literature. However, similar studies should be conducted in different populations and different subtypes of diabetes. Long-term follow-up studies should be conducted in larger populations to better understand the mechanisms underlying this relationship and to determine optimal management strategies for these patients.

## Figures and Tables

**Table 1:** Distribution of lower GIS symptoms of all patients.

Symptoms	Diabetic Group (*n* = 452)	Nondiabetic Group (*n* = 300)	All Patients (*n* = 752)
Chronic constipation, *n* (%)	226 (50)	126 (42)	352 (46.8)
Rectal bleeding, *n* (%)	135 (30)	85 (28.3)	220 (29.2)
Chronic diarrhea, *n* (%)	49 (10.8)	40 (13.3)	89 (11.8)
Weight loss, *n* (%)	42 (9.2)	30 (10)	72 (9.57)

**Table 2:** Comparison of diabetic and nondiabetic groups.

	Diabetic Group (*n* = 452)	Nondiabetic Group (*n* = 300)	*p*
Age (year)	63.4 ± 11.0	62.8 ± 10.8	0.055
Gender, *n* (%)	/	/	0.677
Female	230 (50.9)	148 (49.3)	
Male	222 (49.1)	152 (50.3)	
Hemorrhoidal disease, *n* (%) OR (CI)	214 (47.3)	52 (17.3)	<0.001 * 4.3 (3.0–6.2)
Glucose (mg/dL)	137 (60–480)	89 (49–138)	<0.001 *
eGFR (ml/dak/1,73 m^2^)	82 (7–147)	94 (12–136)	<0.001 *
Hgb (gr/dL)	13.3 ± 2.4	14.3 ± 2.6	<0.001 *
Ferritin (ng/mL)	42 (1–1700)	47 (0–773)	0.880

eGFR, glomerular filtration rate; Hgb, hemoglobin; OR, odds ratio; CI, confidence interval. * *p* < 0.05 is significant. Normally distributed variables were presented as mean ± Standard Deviation, non-normally distributed variables were presented as median (min-max).

**Table 3:** Comparison of hemorrhoid group and non-hemorrhoid group in terms of demographic data.

	Hemorrhoid Group (*n* = 214)	Non-Hemorrhoid Group (*n* = 238)	*p*	Total (*n* = 452)
Gender, *n* (%)			0.530	
Female	109 (50.9)	121 (50.8)		230 (50.9)
Male	105 (49.1)	117 (49.2)		222 (49.1)
Age (year)	63.1 ± 11.0	63.8 ± 10.9	0.607	63.4 ± 11.0
DM duration (year)	13.1 ± 6.9	7.48 ± 4.4	<0.001 *	10.1 ± 6.3
DM Treatment, *n* (%) Only OAD Only insulin	118 (55.1) 14 (6.5)	154 (64.7) 9 (3.8)	0.105	272 (60.2) 23 (5.1)
OAD, *n* (%) Yes No	200 (93.5) 14 (6.5)	229 (96.2) 9 (3.8)	0.203	429 (94.9) 23 (5.1)
Metformine, *n* (%) Yes No	181 (84.6) 33 (15.4)	211 (88.7) 27 (11.3)	0.202	392 (86.7) 60 (13.3)
Insulin, *n* (%) Yes No	96 (44.9) 118 (55.1)	84 (34.9) 154 (65.1)	0.066	179 (39.6) 273 (60.4)
OAD + insuline, *n* (%) Yes No	82 (38.3) 132 (61.7)	75 (31.5) 163 (68.5)	0.129	157 (34.7) 295 (65.3)
HT, *n* (%) Yes No	156 (72.9) 58 (27.1)	139 (58.4) 99 (41.6)	0.002 *	295 (65.3) 157 (34.7)

DM, diabetes mellitus; OAD, oral antidiabetic drug; HT, hypertension. * *p* < 0.05 is significant. Normally distributed variables were presented as mean ± Standard Deviation.

**Table 4:** Comparison of groups in terms of biochemical and hematological parameters.

	Hemorrhoid Group (*n* = 214)	Non-Hemorrhoid Group (*n* = 238)	Total	*p*
Fasting Glucose (mg/dL)	137 (60–358)	137 (60–480)	137 (60–480)	0.669
HbA1C (%)	8.1 ± 2.1	7.6 ± 2.0	7.8 ± 2.0	0.001 *
eGFR (ml/dak/1.73 m^2^)	83.1 (7–147)	81.0 (10–147)	82.4 (7–147)	0.410
Serum Creatinine (mg/dL)	0.86 (0.5–9.6)	0.87 (0.3–6.6)	0.87 (0.3–9.6)	0.549
Hgb (g/dL)	13.5 ± 2.21	13.1 ± 2.58	13.3 ± 2.42	0.165
Ferritin (ng/mL)	38 (2–1093)	48 (1–1700)	42 (1–1700)	0.305
Uric Acid (mg/dL)	5.05.0 (1.9–14)	5.0 (1.7–14.4)	5.0 (1.7–14.4)	0.762
LDL (mg/dL)	122 (38–230)	106 (26–211)	113 (26–230)	0.001 *
TG (mg/dL)	164 (39–817)	136 (38–699)	145 (38–817)	0.005 *
HDL (mg/dL)	40 (11–77)	40 (10–76)	40 (10–77)	0.668

HbA1C, hemoglobin A1C; eGFR, glomerular filtration rate; Hgb, hemoglobin; LDL, low-density lipoprotein; TG, triglyceride; HDL, high-density lipoprotein. * *p* < 0.05 is significant. Normally distributed variables were presented as mean ± Standard Deviation, non-normally distributed variables were presented as median (min-max).

**Table 5:** Comparison of DM duration and blood glucose regulation in the hemorrhoid group according to hemorrhoidal distribution.

	Hemorrhoid Group (*n* = 214)	Age (year)	Diabetes Duration (year)	HbA1C (%)	Glucose (mg/dL)
External hemorrhoid *n* (%)	34 (15.9)	68.7 ± 10.6	10.0 ± 4.8	8.8 ± 1.69	172 (86–357)
Internal hemorrhoid *n* (%) Grade 1 Grade 2 Grade 3 Grade 4	166 (77.6) 115 (53.7) 49 (22.9) 14 (6.5) 2 (0.9)	62.1 ± 10.8	12.9 ± 6.6	7.8 ± 1.9	127 (60–358)
External and internal hemorrhoid, *n* (%)	14 (6.5)	61.1 ± 11.5	23 ± 4.8	9.9 ± 3.7	139 (78–293)
*p*		0.005	<0.001	<0.001	<0.001

HbA1C, hemoglobin A1c.

**Table 6:** Spearman correlation analysis between the parameters and grades of internal hemorrhoids in the hemorrhoid group.

	r	*p*
DM duration, (year)	0.515 (R^2^ = 0.311)	<0.001
HbA1C, (%)	0.161	0.031
Glucose, (mg/dL)	0.055	0.462
LDL, (mg/dL)	0.019	0.798
HDL, (mg/dL)	0.106	0.158
TG, (mg/dL)	0.046	0.543
Hgb, (g/dL)	0.077	0.315
Ferritin, (ng/mL)	−0.172	0.021

DM, diabetes mellitus; HbA1C, hemoglobin A1C; Hgb, hemoglobin; LDL, low-density lipoprotein; TG, triglyceride; HDL, high-density lipoprotein.

## Data Availability

All data generated or analyzed during this study are included in this article. The datasets in the current study are available from the corresponding author upon reasonable request.
